# Discussion before the Sixth Annual Meeting of the Ohio State Dental Society, December 5–6, 1871

**Published:** 1872-03

**Authors:** 


### Proceedings of Societies.

### DISCUSSION BEFORE THE SIXTH ANNUAL MEETING OF THE OHIO STATE DENTAL SOCIETY, DECEMBER 5-6, 1871.

[Continued from page 87.]

EVENING SESSION—QUIZ EXERCISE.

Conducted by Dr. C. R. Butler.

Q. What would you use to devitalize the nerves of the teeth?

A. by a Member. A proportion of morphine and arsenic, or carbolic acid.

Q. How long would you allow the preparations to remain in the teeth?

A. by Dr. Kelsey. I should not like it to remain in over eight hours, if I could avoid it.

Q. What would be the best conditions in a case, to give the most rapid and desirable results?

A. by Dr. Beckwith. I would think the pulp should be exposed, if not entirely open, before the application was made.

Q. How would you proceed to remove the pulp after having been obtunded or devitalized, as spoken of?

A. by a Member. I generally do it with a small instrument of White's.

Q. Would it be necessary to enlarge the orifice, so as to have freer access?

A. by a Member. Yes, sir.

Q. What preparatory treatment would be necessary before filling the canal?

A. by a Member. I would clean it out as thoroughly as possible to the bottom of the fang, perhaps saturate it with a solution of creosote; then I would fill it up solid.

Q. What substance would you use for filling the root?

A. by Dr. Clippinger. If I could get at it, I would fill with gold.

Q. At what age of children or young people would you regard it safe to destroy the pulp and fill permanent teeth?

A. by Dr. Howe. I do not know that I should be governed by the age particularly—that is, if the teeth were filled out.

Q. What is the source of nourishment to the tooth after the pulp is destroyed?

A. by Dr. N. W. Williams. Through the periostium, so far as it is nourished.

Q. Is the crown nourished after the pulp is destroyed?

A. by Dr. Watt. I think not. I think perhaps the dentine at the root receives more or less nourishment, but I do not think the crown receives nourishment.

Q. What would be the ultimate result where the pulp has been destroyed, say from six to thirteen years of age?

A. by Dr. I. Williams. I would think the ultimate result would be that the teeth would be lost. That is, being vascular, and no deposit of calcareous matter taking place, I should think there would be necrosis of the teeth and premature loss.

Q. What would be the conditions where you would think it advisable to destroy the pulp, in young subjects, rather than to extract the teeth at once?

A. by Dr. Berry. If the subject was well put up, stout, and of firm health, there might be a possibility of their keeping them a good while, even after destroying the nerve; but I would always try to take the precaution to control such cases. The operation would very likely prove a failure before a great many years, and the teeth finally have to come out. I have but little confidence in saving such teeth.

Dr. Spellman, by permission, made some remarks in regard to the manner in which some questions had been answered. He did not doubt that the questions may have been answered correctly; but if published to the world, it would seem, from the answers given, that members of the Ohio Dental Society are using arsenic to destroy pulps, as was done ten or twelve years ago. He would not be willing to have such an impression prevail throughout the country with regard to this Society. He thought the best operators in the country would now ignore the use of arsenic entirely. Nothing was so injurious to the teeth as arsenic, causing them to crumble and break down worse than if the pulp is destroyed in any other way. His practice was to put in carbolic acid and creosote, and to repeat these until the pulp is devitalized.

Q. What do you regard as the best way of removing the pulp?

A. by Dr. Buffett. It is better to remove the nerve without any application, if possible.

Dr. Taft, by request, gave his opinion on the subject, remarking that it was his preference to remove it without any application whatever, so far as arsenious acid or any kindred substance was concerned, though he sometimes used carbolic acid, creosote, or something of the kind. But the pulp can be removed, especially from the single root teeth, in a majority of cases, without making any application at all, and perhaps with as little pain to the patient, as would occur if carbolic acid were used. To do this properly, these three precautions must be observed: to have a sufficient opening, a free entrance, and a proper sized and properly formed instrument, sharp, and free from edges. With such an instrument, the obtunding agents would, in almost all cases, be unnecessary. In the buccal roots it was more difficult to remove, and he would seldom remove it from them, but would simply cover it over to protect it.

Q. Don't you think it possible, when you leave a portion of the pulp there, that is not devitalized, for circulation to be established, and that portion remain alive?

A. by Dr. Taft. I know it is possible. I have examined teeth that were treated in that way, and made numbers of experiments with a view to determine it.

Q. Would you expect it to live if you treated the pulp with arsenic?

A. No, never.

### DENTAL MEDICINE.

Dr. Geo Watt, in opening the discussion upon this subject, said that dental medicine involves all that the dentist should know of physiology, pathology, materia medica, therapeutics and chemistry. It involves everything a dentist should know that is strictly dental, except dental mechanism. The foundation is a broad one, but unless we build upon that foundation we fall short of our duty. In treating patients, we should understand everything that would modify that treatment. We must know what the power of life is, whether greater in some directions than others, what the peculiarities of the circulation are, and all such things, before we can treat a patient intelligently. The sex of the patient may have considerable to do in forming a correct estimate of the recuperative energies and vital powers. Members of the profession were too much in the habit of going by guess in prescribing for the diseases they treat.

Dr. J. Taft referred to a little work, issued by Dr. Arthur, of Baltimore, in which he advocates prophylactic measures as preventive of decay. Whether this would be regarded as dental medicine, or dental hygiene; he thought it an important subject. He had examined the work, and considered it a very good little manual on the subject. The arrest of decay in its incipient stages, before filling is required, is, of course, very desirable. He starts out with the idea that the decay of the permanent teeth occurs from the lodgment of foreign substances upon them. His object is, to relieve this contact, or make space between them so as to keep them free from the lodgment of all foreign substances. He advocates the idea of beginning with the first permanent molars, so soon as they appear through the gums, and making a separation by cutting with a file or chisel, between it and the posterior temporary molar, by cutting off a portion of the temporary molar whether decayed or not, but more particularly if decayed. And when the central incisors come in, pursue the same course with them; so that the permanent teeth are kept free from the lodgment of any foreign substances, and so that cleanliness shall be an easy matter. This was a very important point and he had no doubt if this course was pursued it would reduce the amount of proximal decays to a small proportion of what now exists. The attention of dentists, in years gone by, has been given mostly to disease after it has begun and gone on to a considerable extent. Dentists send patients away sometimes, refusing to fill cavities until they become larger and easier to fill. Such should not be the case. Members of the profession ought to study and investigate to ascertain what things deteriorate the teeth; what is liable to do so from the beginning, when the first rudiments are forming, and what may interrupt the perfect development of the teeth. This would involve histology, all the way from the beginning up. The formation of tissues, and everything connected with them, should be investigated, and such things employed as would control and modify them, so as to secure the best possible results. It is time people should awake to the importance of the growth and development of the teeth, and especially those who, to any extent, have anything to do with the subject of medicine and hygiene—all whose attention is directed in any way to the welfare of the body—should feel it their duty to educate the people in regard to these matters, and always and everywhere enter their protests against the vicious habits and customs of life so prevalent and tending so much to break down the race to a mere condition of dwarfage.

### BASES FOR ARTIFICIAL TEETH.

Dr. W. M. Herriott, in opening this discussion, said that about two years since, Dr. Watt reported that he had under consideration and experiment, at that time, a material as a base for artificial teeth, and a year since he was able to tell us what he had accomplished with this material, and that he was then ready to offer it to the profession as a base for artificial teeth. Dr. Herriott said some objections to this base, by other dentists, had come to his knowledge, though he had found but little trouble with it himself. Some have objected that it was too heavy. That was a very slight objection to him, as he had been able to construct it without giving it too much weight. He did not find it as liable to objection as many other artificial bases, of low priced materials, they were sometimes compelled to use.

Dr. Watt remarked that so far as weight was an objection, he did not know how to overcome that. He did not see how he could reduce his own weight without reducing his strength, and he thought it was about the same way with this base. While the weight may be an objection sometimes, it is just as often an advantage. He had seen patients who could not for years masticate their food, when, by simply making them a heavy set of plates, they were able to masticate with ease. The operator must be as careful with regard to contamination of the metal as he would be of gold. He had known some cases where dark spots had appeared upon it, and a few cases where there were brittle spots. It may, perhaps, have been for want of care in compounding, in the first place. He did not pretend to be infallible, though he took a great deal of pains. In every case where he had been able to trace spots or discoloration, or brittle spots had appeared, it had been where the vessel in which it was melted had been used for melting other materials. In such instances he had found the presence of other metals entirely foreign to it, and had cut them out.

Again, many do not realize how thin they can cast plates with this material, and almost always make them too heavy, especially the upper plates. If there is not an accurate fit, the weight will be a very serious objection; if, however, there is a good fit, the weight will not be so objectionable, but will often answer better than a plate of less weight.

Dr. Kelsey had no doubt that Dr. Watt's was a good metal, but he desired that he should make known what it was composed of. He used a material for a base, himself, that he found good, and he had no objection to telling what it was. He used block tin, purified as much as possible, with one sixtieth part of bismuth, simply to give it polish.

Dr. Warner had used Dr. Watt's base, from the time it was first introduced, as long as he did any mechanical work. He believed the objections against it arise from the manner of its manipulation; probably in ninety-nine cases out of one hundred it is made too heavy when weight is offered as an objection. Sometimes, however, the weight was a benefit. Those who use rubber are sometimes under the necessity of loading their plates with some heavier material. If properly manipulated, he thought it was superior to any cheap base he had used, and he had very little sympathy with those who were insisting upon the necessity of using rubber, and paying fifty dollars for a license in order to use it. He thought it unreasonable to ask Dr. Watt to make known the ingredients of which his material was composed. If there was anything to be made from it, certainly Dr. Watt, in consideration of his long labors in the profession, and the vast amount of instruction given them gratuitously, should receive due compensation for his discovery. He did not believe, if it was made known, that it could be manufactured much cheaper than the Doctor was selling it himself.

Dr. Herriott thought there was little danger of getting too much weight in the lower plates. He referred to the use of springs in double sets in early times, to produce a pressure upon the lower structure. He believed with this material of

Dr. Watt's, he could construct a base that would be as clean and pure in the mouth as anything he had ever used. He said it would adhere to porcelain with the tenacity almost of any two substances you can unite.

Dr. Clippinger said he had been working with this base of Dr. Watt's a little over a year. He had gradually changed from one base to the other in his practice, and, quitting rubber, had adopted the other material. If dentists wish, they can gradually change from one base to another. Most persons who come to dentists for treatment suppose that they will know what they need, and they should recommend what they think best. If they do not think you know best, the quicker you get rid of them the better. He found this material better than anything he had used, combining strength and solidity, easily kept clean, and satisfactory to the wearer, when properly constructed.

Dr. Rosson said, if he were to be confined to the one material he considered best of all others for a base, he would prefer gold. Next to that he would take Dr. Watt's material. In using the gold plate, he would want the privilege of using a rubber attachment, so that he could make the gold plate very thin and light, and make a perfect fit without springing the plate.

Dr. J. Taft thought perhaps most of the claims made for the different bases for artificial teeth were true, yet there is nothing that is applicable to all cases and conditions. Almost everything that has been introduced is susceptible of being advantageously used in cases to which they are adapted, and he thought wisdom would dictate to the profession that they should gather up all these and have them at their command, whenever they would be best adapted. Every person who will open his eyes and observe closely, will admit that rubber is not fit to be used in some cases. That material should be selected which will best suit the patient and be most free from producing injurious effects, in each individual case.

Dentists had departed, to a great extent, from the mechanical part of the profession, and committed it to the hands of mechanics. He would put nothing into the mouths of patients that will manifestly do injury. This he believed to be the only true course. He was well convinced that rubber did a great deal of injury in many cases; probably in nine cases out of ten, where rubber plates are used, there is an abnormal condition of the mucous follicles and consequent irritation. Extreme caution should be exercised in regard to the use of plates of this material, or anything else that is likely to cause a disturbance of normal conditions.

### DISCUSSION ON DR. SMITH'S PAPER ON "MECHANICAL DENTISTRY—ITS DECLINE AS AN ART."

Dr. Rehwinkle said he regretted as much as the author of the paper that dentists, throughout the United States, are contented simply with the manufacture of porcelain work—of which he thought very highly. If that could be made as easily as rubber, he thought it would emancipate the profession from the use of teeth manufactured by others, and would establish them as artists, and give them that individuality which every painter and sculptor impresses upon his work. But, as it now is, most operators feel compelled to give their energy and strength to operative dentistry, and abandon the mechanical field altogether; and the manufacture of teeth is left to those who, in many cases, have no higher aim than the manufacturer of boots and shoes.

Dr. C. R. Butler thought it too true that the department of mechanical dentistry had greatly declined among them. Those of them that had a love for fitting out artificial dentures had nearly abandoned it and lost their love for it. He thought dentists should be able to produce a set of teeth that could be mounted on gold, or any other material, so as to give an individuality to it and stamp it with his skill. He had some specimens of block and single teeth, made years ago, that he kept as a sort of curiosity, and was beginning to value them almost as relics of a lost art.

Dr. A. Berry referred to a number of old specimens that he had carefully preserved, which showed the greatest artistic skill in their manufacture.

Dr. Smith said some time since a lady had come to a dentist in Cincinnati, to get a set of teeth, and the lady, not liking the teeth altogether, the dentist said to her, "Why, madam, those are the latest style, and have only been out a short time. [Laughter.] I have only been able to get a few of this latest style." The people, said he, are being educated to appreciate a set of teeth as they would a new carpet or fashionable furniture. The habit of putting the same kind of teeth in everybody's mouth had tended much to bring this about. The teeth, as manufactured at the present day, are too regular to be beautiful and to have a natural appearance. An operator should never make two sets of teeth just alike.

Dr. Rehwinkle related a couple of amusing cases that had come up in his practice. The latter was that of an old lady, between sixty and seventy years of age, who came to him several years ago, for whom he made a full denture. She was a lady of dark complexion, gray hair, and bore the marks of age. He requested that she should leave him to exercise his judgment in regard to the selection and manufacture of the set of teeth for her which would be best suited to her age and present a natural appearance. After a great deal of work and pains spent upon them, he produced a set which he thought admirably adapted for the person for whom they were intended. The old lady, however, upon coming to the office for the teeth, was very much displeased with them, and told him she was willing to pay her money, but she "wanted pretty teeth." He tried in vain to reason the case with her, and to get her to wear them and get the opinion of her friends in regard to them. She desired nice little white teeth, such as she had seen young ladies wearing.

After several visits to the office to complain about the teeth, he finally prevailed upon her to keep them, by letting her have them at a reduced price—the old lady afterward boasting how smart she had been in getting her teeth so cheaply.

Dr. Rosson thought he had heard members of the Society, in former years, argue that the business of the profession should be divided, and thought they did not wish to attend to the mechanical department. This, he thought, had a tendency to drive it into the channel they now deplored.

Dr. Herriott was glad the subject had been brought up for discussion. He hoped the question would be brought out in such a light as that the report of the meeting would show they were not in favor of setting aside this important branch of the profession, for which a great deal more money is paid than for surgical dentistry. The largest proportion of the amount received by the four hundred dentists in the State is received for mechanical dentistry.

Dr. Templeton, corresponding member from the Pennsylvania State Dental Society, said he had been particularly pleased with the turn the discussion had taken. Some two or three years ago he had a conversation on this same subject with an operator in Philadelphia, a man who still continues to carve out teeth and mount them on gold plates. This man told him that some time previous to that, a lady, the wife of a portrait painter, had received a set of teeth from some man who made cheap work; but this artist was so dissatisfied with them that he came to this dentist and told him that the expression of the face was all wrong, and wanted him to try and see if he could not restore the natural expression of the countenance, and pointed out where the set that had been made was defective. This man made a model, and placing it in the mouth of the lady, had the portrait painter to show wherein it was still deficient, and to suggest any improvements he saw it needed. He did so, pointing out where it needed trimming a little and where it required filling up, &c. He then went to work and produced a set of teeth so completely coming up to the painter's ideas of what they should be to restore the natural expression, as to entirely satisfy him with the result. He believed this illustrated what should be the work of the dentist in order to fulfill his mission.

Dr. Butler wished that all would take note of the reference made by Dr. Smith in regard to students who are taken into offices to learn dentistry. They are taught nothing but rubber; find these young men where you will in search of a place, and ask them what they can do, they will reply: "I can put up rubber plate;" but they are ignorant of other branches of the work of the profession. They should be thoroughly trained in all departments of the business.

Dr. Watt remarked that the block work of dentists was not right, because not done upon the right plan. Some of the old block work is magnificent. He regretted having parted, in his generosity, with some beautiful specimens made forty years ago. Unless they lifted the department of mechanical dentistry from its present position, they might almost as well surrender the fort and retire. Though he feared dentists could not compete with the traveling quacks who sold sets of teeth at ten dollars, yet he suggested that, when patients came to them for a set of teeth, that they take great care in getting models fitting accurately, and indicating everything needed to give them such a natural appearance as would be suited to that particular patient, so that the dentist, if he sends it off to a block workman, will be able to do his work more intelligently. Before carved work can be brought to that state of perfection, however, every dentist must be enough of an artist to meet these requirements fully.

